# 

**Published:** 1812-08

**Authors:** 


### MONTHLY CATALOGUE OF MEDICAL BOOKS.

AN Address to the Apothecaries of Great Britain; with an Appeal to the Committee to whom the Interests of Pharmacy are delegated, by the general Meeting held at the Crown and Anchor Tavern, London, July 3d, 1812. By Pharmacopola Verus. 8vo. —Sherwood and Co.

On the uncertainty of the Signs of Murder in the case of Bastard Children. By the late William Hunter. 8vo.—Callow.

Treatise on the Influence of Climate on the Human Species; and on the varieties of Men resulting from it; including an account of the criteria of Intelligence which the form of the Head presents, and a sketch of a rational System of Physiognomy, as founded on Physiology. By N. C. Pitta, M.D. 8vo.—Longman and Co.

Account of the Island of Madeira. By N. C. Pitta, M.D. 8vo. —Longman and Co.

Elements of Chemical Philosophy. By H. Davy. Vol. 1. 8vo. —Johnson and Co.

---

### *NOTICES TO CORRESPONDENTS.*

*Communications are received from Dr. Wilmer, Dr. Clarke, Mr. Murray, &c. &c. &c. which are intended for insertion in our next number.*

*The Case of* Hernia Cerebri, *with a Drawing, communicated from the Blenheim Street Medical Society, will also be given to the public as soon as possible, with an Engraving.*

*The Paper on Animal Poisons is under consideration; and, after some small alterations made under the permission of the ingenious author, will be inserted in our Journal at the earliest opportunity.*

